# A History of the British Fresh-Water Algæ

**Published:** 1846-01

**Authors:** 


### A History of the British Freshwater Algæ. By *Arthur Hill Hassall*, F.L.S. &c. 2 vols. 8vo. Highley and Baillière.

Countless as are the works which daily and almost hourly teem from the press, the number of those which justly lay claim to originality, and which in any considerable degree advance the knowledge of the subject of which they treat, is proportionally small indeed. To this small number of original publications, that now before us pre-eminently belongs, it abounding in records of new and curious facts and productions.

The department of Natural Science, to the elucidation of which the History of the British Freshwater Algæ is devoted, while it has ever been considered to be one of the most interesting, was notoriously, up to the period of its publication, one of the least explored, and in consequence least understood, portions of our Flora, "the works and memoirs," to adopt the language of the author, "comparatively few in number which had then appeared, abounding with descriptions incomplete, inaccurate, or repetitions of the same productions and facts under different forms and appearances."

The comparative neglect with which the vegetation of our freshwaters has until recently been treated was most undeserved, for, while many of the species themselves are amongst the most beautiful objects in nature, the study of them all is of the highest interest and importance to the physiologist; for it is through these simply organised productions that we must first hope to arrive at a perfect knowledge of the all-important processes of Nutrition, Growth, and Reproduction, for it is in them that those processes are carried on with the fewest accessories, and with the least complication. Under each of these heads, and especially upon that of Reproduction, a number of highly curious observations are recorded.

The Freshwater Algæ are also rendered interesting by reason of the very opposite opinions and discussions to which they have given rise, one class of observers regarding them as animal productions, and another as subjects of the vegetable kingdom. The truth in this instance, as in many others, would appear to lie in the mean, thus it is probable that in certain of their attributes they are vegetable, while in others they are animal; in other words, that they form a close connecting link between the two arbitrarily constituted kingdoms of the organic world. The terms "animal" and "vegetable," when used to designate the higher forms of organization, are well understood, and to these they can scarcely be mis-applied. As we descend, however, to the lower types of organic life, the greatest difficulty, a difficulty often insuperable, is experienced in deciding upon the true position in either kingdom which many productions ought to occupy. All attempts at a strict definition, which, on the one hand, shall include every animal, and, on the other, every vegetable, have hitherto signally failed. It is more consonant with reason and with our present information to conclude that no exact line of demarcation exists, and that the one organic division merges insensibly into the other.

The double attributes which pertain to many of the Algæ, will be evident, in some degree, from the following quotation.

"At length the granules become perfected, and they are now seen moving restlessly about the interior of the cell, frequently striking against its walls, as though anxious to escape from the confinement of their narrow cell, and to rove about, independent beings, through the waters, in search of an appropriate abiding-place. Having escaped from the cells, which they are enabled to do, not as Agardh supposed, by the multiplied knockings of their beaks against its sides, whereby its fibres become displaced, but either by rupturing its walls, through their increased development, as in *Lyngbya*, &c., or by some special provision, as in *Vesiculifera*, *Zygnema*, &c., they fall into the water, through which they speedily begin to move hither and thither; now progressing in a straight line with the rostra in advance; now wheeling round and pursuing a different course; now letting their rostra drop, and oscillating upon them, like (to compare small things with great) balloons ere the strings are cut, or like tops, the centripetal force being nearly expended; now altogether stopping, and anon resuming their curious and eccentric motions. Truly wonderful is the velocity with which these microscopic objects progress, their relative speed far surpassing that of the fleetest race-horse. After a time, however, which frequently extends to some two or three hours, the motion becomes much retarded, and at length, after faint struggles, entirely ceases, and the zoospores then lie as though dead: not so, nevertheless; they have merely lost the power of locomotion; the vital principle is still active within them, and they are seen to expand, to become partitioned, and, if the species be of an attached kind, each zoospore will emit from its transparent extremity two or more radicles, whereby it becomes finally and for ever fixed. Strange transition, from the roving life of the animal to the fixed existence of the plant! In exact correspondence with this, is what occurs with the Zoophytes."

One of the most singular of the many interesting particulars described in the work, is the fact of the colouration of vast extents of water by means of several species of exceedingly minute Algæ, different species imparting different tints. Thus ponds, lakes, reservoirs, and even portions of the ocean itself often exhibit various bright colours, occasioned by the presence of some minute Algæ diffused through the water. The Red Sea is now known to derive its name from an Alga of a blood-red colour, which periodically manifests itself in its waters. This discovery was first made by Ehrenberg, so far back as 1819, but his account of it was entirely overlooked until after the publication of the letter of M. Evenor Dupont, addressed to his friend M. Isidore Geoffroy-Saint-Hilaire, in which he thus describes the extraordinary phenomenon of the coloration of the waters of the Red Sea.

"You demand of me certain details in reference to the circumstances in which I gathered the *Cryptogamic* plant which I sent you from the Red Sea, and which you told me appeared a new species. They are as follow:—

"The 8th July last (1843) I entered into the Red Sea by the Strait of Babelmandel upon the steam-boat the Atalanta, belonging to the Indian Company. I demanded of the captain and the officers, who for a long time navigated in these latitudes (parages) what was the origin of this ancient name of the Red Sea; if it was owing, as some have pretended, to sands of that colour, or, according to others, to rocks. None of these gentlemen could reply to me; they never, they said, remarked anything to justify this denomination. I observed then for myself as we advanced: whether the ship approached by turns the Arabian coast or the African coast, the red was in no part apparent. The horrid mountainous barriers which border the two banks were uniformly of a blackish brown, except where in some places the appearance of an extinct volcano had left long white streams. The sands were white, the reefs of coral were white also; the sea of the most beautiful cærulean blue. I had given up the hope of discovering my etymology.

"On the 15th of July the burning sun of Arabia awoke me suddenly by shining all at once, from the horizon without spot, and in all its splendour. I turned myself mechanically towards the window of the poop to seek a remnant of the fresh air of the night before the ardour of the day had devoured it. What was my surprise to behold the sea tinted with red as far as the eye could reach! Behind the ship, upon the deck, and on all sides, I saw the same phenomenon.

"I interrogated the officers anew. The doctor pretended that he had already observed this fact, which was, according to him, produced by the fry of fish floating on the surface; the others said that they did not recollect having seen it before. All seemed surprised that I should attach such interest to it.

"If it be necessary to describe the appearance of the sea, I should say that its surface was covered with a compact stratum of but little thickness, but of a fine texture, of a brick red, slightly tinged with rouge; sawdust of this colour, of mahogany, for example, would produce very nearly the same effect. It seemed to me, and I said at the time, that it was a marine plant. No one seemed of my opinion; so with a pail tied at the end of a rope I was able to gather, with one of the sailors, a certain quantity of the substance: this with a spoon I introduced into a white glass bottle, thinking that it would be the better preserved. The next day the substance had become of a deep violet, and the water had taken a pretty pink tinge. Fearing that the immersion would hasten the decomposition instead of preventing it, I emptied the contents of the bottle upon a piece of cotton (the same which I remitted to you). The water passed through it and the substance adhered to the tissue. In drying it became green, as you actually saw it. I ought to add, that on the 15th of July we were by the side of the town of Cosseir; that the sea was red the whole day; that the next, the 16th, it was the same until near mid-day, the hour at which we found ourselves before Tor, a little Arabian village, the palms of which we perceived in an oasis on the border of the sea, below the chain of mountains which descends from Sinai, even to the sandy shore. A little after mid-day, the 16th, the red disappeared, and the surface of the sea became blue as before. The 17th we cast anchor at Suez. The red colour had consequently showed itself from the 15th of July, towards 5 o'clock in the morning, up to the 16th, nearly an hour after mid-day; that is to say, during thirty-two hours. During this interval the steam-boat, making eight knots an hour, as said the sailors, had traversed a space of 256 miles, or 85 leagues and a third.

"In the different works relative to Egypt and the Red Sea which I have had occasion to read, I do not recollect to have found mention made of a similar fact: it appears to me, nevertheles, but little probable that it has not been observed by others. I reproached myself for not having questioned the Arabian pilot whom we had on board, and who for twenty years traversed that sea. This idea unhappily presented itself too late.

"If it should be in your opinion worth the pains, I would demand new observations of the surgeon or officers of the Atalanta, for it would be easy for me to write to them by way of Alexandria."

Charlatans, calculating in advance the periodical returns of the phenomon, were thus enabled to terrify and govern the populace, by pronouncing its appearance to be the certain forerunner of some dire calamity.

The uses of the Confervæ Mr. Hassall treats as four-fold; three only of these uses we shall here notice.

"The second purpose to which the *Confervæ* are subservient is one of great importance, being the purification of the fluid in which they dwell, laden, as it frequently is, with various deleterious gases, arising from the death and decomposition of various animal and vegetable substances; thus deriving their own origin, for the most part, in the midst of impurity, they are the agents employed in removing this impurity, which salutary office they perform in the following way. Amongst the most noxious of these gases to animal life are carbonic acid and carburetted hydrogen; now carbon, the base of these, constitutes the pabulum, or food, of plants. These two gases, then, the *Confervæ* decompose, retaining the carbon for their own support, and setting free the oxygen and hydrogen; thus, not merely decomposing and removing what is hurtful, but restoring to the water oxygen, the essential to all animal life whether found in air or water. Seeing, then, the important purpose which these apparently frail and insignificant productions fulfil, who is there who would venture to remove even this one small and remote link from the chain of Nature's works; and would be answerable for the consequences of its removal? Who can tell what baneful influence might not arise, and spread disease and death through whole districts? a calamity which, even as things are now ordained, is occasionally permitted to overtake us. Should any individual be sceptical as to the influence of these productions, and whether a respiration of the kind I have alluded to, and attended with the same results, really occurs, let him put into a tumbler of water a little of the first *Conferva* which he may meet with in his next ramble, and, placing it in the rays of the sun, watch it for a short time; he will soon observe globules of a gas, at first small, but soon becoming larger, to collect upon the surface of the filaments, which, when they have attained a sufficient size, will quit their attachment, rise to the surface of the water, and at last lose themselves in the surrounding air. This will, I think, satisfy him that a respiration of some kind, is carried on; and should he wish to ascertain the nature of the gas thus eliminated, whether it be really oxygen or not, this may be done by procuring a considerable quantity of any floating species of *Conferva*, and placing it in a trough of water, over which should be put a glass jar also filled with water, having an air-tight collar adapted to it, so disposed as to catch the gaseous globules as they ascend. As soon as the glass jar becomes filled with the gas, let the air-tight collar be removed and a piece of ignited phosphorus be quickly plunged into the interior of the jar, when the brilliant and dazzling combustion which will instantaneously ensue will afford a proof conclusive of the nature of the elimination. The honour of this discovery, if it can be deemed one, for it is but the extended application of the common principle of the respiration of plants generally, is in this country attributed to Priestley; but so obvious is it that it scarcely required the penetration of a mind like his for its detection: Vaucher alludes to it cursorily.

"The third use of the *Confervæ* is a moral one. Every created thing, rightly viewed, is capable of imparting this moral lesson, be it the kingly lion or the spurned reptile; the beautiful and scented flower, or the more humble productions which have been engaging our attention. There is no imperfection acknowledged in nature, nor are there, strictly, degrees of comparison; everything is superlative, is best and perfect from the hands of God who made it, alike unsurpassable and inimitable.

"Then, lastly, there is the intellectual benefit derived by those who study this or any branch of Nature's works. There are those who regard the pursuits of natural history as trivial and tending to no useful purpose; but these are but superficial observers, with hearts and minds alike incapable of appreciating the depths and hidden beauties of the study. I maintain, in opposition to these, that there is room in the contemplation of, and search after, the laws and phenomena of animal and vegetable life and growth, for the exercise of an enlarged and enlightened understanding."

Elsewhere, Mr. Hassall expresses his conviction, that a good and useful paper might be made out of various species of Confervæ. It would be well that the trial should be made, as it might lead to some very important results.

Some idea of the originality of the History of the British Freshwater Algæ may be gathered from the following statement. In the most recent work on the Algæ of this Country, both marine and freshwater, 165 species are described as inhabitants of our fresh-waters; while, in the work under review, no less than 467 productions are figured and characterized. The number of species included in the genus Zygnema up to the date of Mr. Hassall's publication were 5, in that gentleman's volumes 43 are described.

An excellent feature in the work before us is the constant and copious reference which is made to the writings of various French and German naturalists, in which respect English writers are usually very deficient.

The style of the work is as popular and as pleasing as the nature of the subject admits of. There is a lengthened and valuable Introduction, in which the subjects of Growth, Nutrition, and Reproduction are treated *generally*, as well as under the different headings into families, lesser chapters, in which these processes are described more *in detail*. The entire volume of letter-press is, moreover, enlivened by the insertion of numerous striking and beautiful passages taken from the writings of some of the older naturalists, Linnæus, Ray, Haller, &c.

The volume of Plates, which are coloured, were executed by the author himself, a guarantee for their accuracy.

In conclusion, we would observe of Mr. Hassall's laborious and curious work, that it supplies a very great desideratum, not merely in the natural history of this country, but of the world at large, and that, from the circumstance of the Algæ forming so close a connecting link between the animal and vegetable kingdoms, one not less necessary to the Zoologist than the Botanist.

